# Identification of trema in first episode psychosis: a case report

**DOI:** 10.1192/j.eurpsy.2022.2040

**Published:** 2022-09-01

**Authors:** C. Alvarez Garcia, D. García Martínez, L. Nocete Navarro

**Affiliations:** 1 Hospital Universitario Príncipe de Asturias, Psychiatry, Alcalá de Henares, Spain; 2 Hospital Universitario de La Paz, Psichyatry, Madrid, Spain

**Keywords:** trema, Anxiety, Psychosis, anguish

## Abstract

**Introduction:**

The concept of trema refers to an initial phase in the psychotic process that is characterized by intense anguish, an experience of hostility and a feeling of imminent catastrophe. This situation engenders in the patient a sensation of being in a tunnel than can only lead to something terrible but ineffable.

**Objectives:**

To illustrate the incipient phase of psychotic disorder though the presentation of a case.

**Methods:**

A presentation of a clinical case.

**Results:**

A 29-year-old man attends the emergency department due to anxiety of one moth of evolution, that had debuted after a stressful event in the patient’s life such as loss of employment. He suffered from intense morning-predominance anguish, depersonalization episodes, insomnia, hallucinosis, cognitive blocks that occasioned him great anxiety and apragmatic behaviors. Besides, he had language alteration and autolytic ideation with previous autolytic gestures. After evaluation, he was diagnosed with psychotic episode. He was hospitalized, and treatment with olanzapine and lorazepam was started.

**Conclusions:**

With the exhibition of this case, we intended to point up the importance of a differential diagnosis with different disorders marked by anxiety as the main symptom. In our case, panic disorder should be taken in account as a differential diagnosis. Furthermore, as the evidence shows, the identification of prodromic phases in schizophrenia allows an early diagnosis and early intervention, improving the prognosis.

**Disclosure:**

No significant relationships.

